# Supplementary material for: Genetic Correlation of miRNA Polymorphisms and STAT3 Signaling Pathway with Recurrent Implantation Failure in the Korean Population
Source: Int J Mol Sci. 2023 Nov 27;24(23):16794. doi: 10.3390/ijms242316794 (PMC10706094; doi:10.3390/ijms242316794)
Supplement: Supplementary file 1 [file ijms-24-16794-s001.zip › ijms-2719710-supplementary.pdf]

Supplementary Table S1. Allele combinations analysis for miRNA polymorphisms in RIF patients and controls

Allele combinations	Controls (2n=536)	Cases (2n=322)	OR (95%CI)	*P*
miR-218-2 rs11134527 G>A/miR-34a rs2666433 G>A/miR-34a rs6577555 C>A/miR-130a rs731384 G>A				
A-G-C-G	154 (28.7)	85 (26.7)	1.000 (reference)	
A-G-C-A	30 (5.6)	10 (3.1)	0.615 (0.286-1.319)	0.208
A-G-A-G	57 (10.6)	48 (15.1)	1.552 (0.972-2.478)	0.065
A-G-A-A	9 (1.7)	2 (0.6)	0.410 (0.086-1.941)	0.339
A-A-C-G	66 (12.3)	32 (10.1)	0.894 (0.542-1.473)	0.659
A-A-C-A	4 (0.7)	8 (2.5)	3.687 (1.078-12.61)	**0.034**
A-A-A-G	3 (0.6)	0 (0.0)	0.263 (0.013-5.149)	0.553
G-G-C-G	96 (17.9)	54 (17.0)	1.037 (0.676-1.59)	0.868
G-G-C-A	8 (1.5)	11 (3.5)	2.535 (0.981-6.55)	**0.048**
G-G-A-G	43 (8.0)	24 (7.5)	1.029 (0.584-1.813)	0.922
G-G-A-A	7 (1.3)	9 (2.8)	2.370 (0.852-6.596)	0.090
G-A-C-G	48 (9.0)	37 (11.6)	1.421 (0.857-2.355)	0.172
G-A-C-A	11 (2.1)	0 (0.0)	0.080 (0.005-1.374)	**0.018**
G-A-A-G	0 (0.0)	2 (0.6)	9.192 (0.436-193.8)	0.127
miR-218-2 rs11134527 G>A/miR-34a rs2666433 G>A/miR-34a rs6577555 C>A				
A-G-C	182 (34.0)	92 (28.9)	1.000 (reference)	
A-G-A	65 (12.1)	51 (16.0)	1.561 (1.001-2.433)	**0.049**
A-A-C	71 (13.2)	42 (13.2)	1.177 (0.745-1.857)	0.485
A-A-A	3 (0.6)	0 (0.0)	0.283 (0.014-5.549)	0.553
G-G-C	105 (19.6)	67 (21.1)	1.269 (0.854-1.886)	0.237
G-G-A	51 (9.5)	33 (10.4)	1.287 (0.777-2.132)	0.326
G-A-C	59 (11.0)	35 (11.0)	1.180 (0.725-1.921)	0.506
G-A-A	0 (0.0)	2 (0.6)	9.919 (0.471-208.9)	0.114
miR-218-2 rs11134527 G>A/miR-34a rs2666433 G>A/miR-130a rs731384 G>A				
A-G-G	207 (38.6)	132 (41.5)	1.000 (reference)	
A-G-A	40 (7.5)	13 (4.1)	0.515 (0.265-0.998)	**0.046**
A-A-G	70 (13.1)	32 (10.1)	0.724 (0.452-1.16)	0.178
A-A-A	4 (0.7)	8 (2.5)	3.167 (0.935-10.73)	0.071
G-G-G	140 (26.1)	78 (24.5)	0.882 (0.62-1.255)	0.485
G-G-A	15 (2.8)	20 (6.3)	2.111 (1.044-4.269)	**0.034**
G-A-G	49 (9.1)	39 (12.3)	1.260 (0.785-2.024)	0.338
G-A-A	11 (2.1)	0 (0.0)	0.069 (0.004-1.177)	**0.008**
miR-218-2 rs11134527 G>A/miR-34a rs6577555 C>A/miR-130a rs731384 G>A				
A-C-G	216 (40.3)	116 (36.5)	1.000 (reference)	
A-C-A	36 (6.7)	19 (6.0)	0.987 (0.542-1.799)	0.967
A-A-G	60 (11.2)	47 (14.8)	1.465 (0.941-2.283)	0.090
A-A-A	9 (1.7)	3 (0.9)	0.624 (0.166-2.349)	0.555
G-C-G	148 (27.6)	90 (28.3)	1.138 (0.805-1.607)	0.465
G-C-A	17 (3.2)	11 (3.5)	1.210 (0.549-2.671)	0.636
G-A-G	43 (8.0)	28 (8.8)	1.218 (0.719-2.063)	0.462
G-A-A	7 (1.3)	8 (2.5)	2.138 (0.756-6.045)	0.143
miR-34a rs2666433 G>A/miR-34a rs6577555 C>A/miR-130a rs731384 G>A				
G-C-G	250 (46.6)	136 (42.8)	1.000 (reference)	
G-C-A	38 (7.1)	22 (6.9)	1.052 (0.598-1.852)	0.860
G-A-G	98 (18.3)	72 (22.6)	1.354 (0.937-1.956)	0.106
G-A-A	17 (3.2)	10 (3.1)	1.069 (0.476-2.4)	0.871
A-C-G	115 (21.5)	67 (21.1)	1.075 (0.746-1.549)	0.699
A-C-A	14 (2.6)	8 (2.5)	1.168 (0.493-2.77)	0.724
A-A-G	4 (0.7)	3 (0.9)	1.363 (0.301-6.181)	0.703
miR-218-2 rs11134527 G>A/miR-34a rs2666433 G>A				
A-G	247 (46.1)	144 (45.3)	1.000 (reference)	
A-A	75 (14.0)	41 (12.9)	0.938 (0.608-1.445)	0.771
G-G	156 (29.1)	99 (31.1)	1.089 (0.786-1.507)	0.609
G-A	58 (10.8)	38 (11.9)	1.124 (0.711-1.776)	0.617
miR-218-2 rs11134527 G>A/miR-34a rs6577555 C>A				
A-C	253 (47.2)	134 (42.1)	1.000 (reference)	
A-A	69 (12.9)	51 (16.0)	1.396 (0.919-2.12)	0.118
G-C	164 (30.6)	102 (32.1)	1.174 (0.849-1.624)	0.331
G-A	50 (9.3)	35 (11.0)	1.322 (0.818-2.136)	0.254

miR-218-2 rs11134527 G>A/miR-130a rs731384 G>A				
A-G	276 (51.5)	163 (51.3)	1.000 (reference)	
A-A	46 (8.6)	22 (6.9)	0.810 (0.47-1.395)	0.446
G-G	191 (35.6)	118 (37.1)	1.046 (0.775-1.413)	0.769
G-A	23 (4.3)	19 (6.0)	1.399 (0.739-2.647)	0.301
miR-34a rs2666433 G>A/miR-34a rs6577555 C>A				
G-C	288 (53.7)	160 (50.3)	1.000 (reference)	
G-A	115 (21.5)	83 (26.1)	1.299 (0.923-1.829)	0.133
A-C	129 (24.1)	76 (23.9)	1.060 (0.753-1.495)	0.737
A-A	4 (0.7)	3 (0.9)	1.350 (0.298-6.11)	0.705
miR-34a rs2666433 G>A/miR-130a rs731384 G>A				
G-G	348 (64.9)	210 (66.0)	1.000 (reference)	
G-A	55 (10.3)	33 (10.4)	0.994 (0.625-1.582)	0.981
A-G	119 (22.2)	71 (22.3)	0.989 (0.704-1.389)	0.948
A-A	14 (2.6)	8 (2.5)	0.947 (0.391-2.296)	0.904
miR-34a rs6577555 C>A/miR-130a rs731384 G>A				
C-G	365 (68.1)	205 (64.5)	1.000 (reference)	
C-A	52 (9.7)	31 (9.7)	1.061 (0.659-1.71)	0.806
A-G	102 (19.0)	76 (23.9)	1.327 (0.942-1.869)	0.106
A-A	17 (3.2)	10 (3.1)	1.047 (0.471-2.33)	0.910

Note: 95% CI, 95% confidence interval; OR, odds ratio; N/A, not applicable.

Supplementary Table S2. Combined genotype analysis for miRNA polymorphisms in RIF patients and controls

Genotype combinations	Controls (n=268)	RIF patients (n=161)	AOR (95% CI)*	*P*
miR-218-2 rs11134527 G>A/miR-34a rs2666433 G>A				
AA/GG	58 (21.6)	29 (18.0)	1.000 (reference)	
AA/GA	30 (11.2)	19 (11.8)	1.302 (0.626-2.709)	0.480
AA/AA	6 (2.2)	1 (0.6)	0.339 (0.039-2.961)	0.328
AG/GG	72 (26.9)	44 (27.3)	1.237 (0.689-2.22)	0.476
AG/GA	53 (19.8)	40 (24.8)	1.507 (0.821-2.767)	0.186
AG/AA	9 (3.4)	3 (1.9)	0.692 (0.17-2.814)	0.607
GG/GG	23 (8.6)	15 (9.3)	1.328 (0.598-2.948)	0.486
GG/GA	14 (5.2)	8 (5.0)	1.193 (0.444-3.209)	0.727
GG/AA	3 (1.1)	2 (1.2)	1.887 (0.276-2.909)	0.518
miR-218-2 rs11134527 G>A/miR-34a rs6577555 C>A				
AA/CC	58 (21.6)	26 (16.1)	1.000 (reference)	
AA/CA	33 (12.3)	18 (11.2)	1.211 (0.578-2.536)	0.611
AA/AA	3 (1.1)	5 (3.1)	3.54 (0.777-6.128)	0.102
AG/CC	77 (28.7)	50 (31.1)	1.419 (0.79-2.551)	0.242
AG/CA	51 (19.0)	30 (18.6)	1.27 (0.662-2.436)	0.472
AG/AA	6 (2.2)	7 (4.3)	2.603 (0.793-8.549)	0.115
GG/CC	26 (9.7)	14 (8.7)	1.246 (0.557-2.785)	0.592
GG/CA	11 (4.1)	8 (5.0)	1.648 (0.591-4.595)	0.340
GG/AA	3 (1.1)	3 (1.9)	1.739 (0.305-9.923)	0.533
miR-218-2 rs11134527 G>A/miR-130a rs731384 G>A				
AA/GG	67 (25.0)	40 (24.8)	1.000 (reference)	
AA/GA	26 (9.7)	8 (5.0)	0.531 (0.219-1.292)	0.163
AA/AA	1 (0.4)	1 (0.6)	1.375 (0.083-2.827)	0.824
AG/GG	104 (38.8)	63 (39.1)	1.017 (0.616-1.68)	0.947
AG/GA	29 (10.8)	22 (13.7)	1.402 (0.699-2.814)	0.342
AG/AA	1 (0.4)	2 (1.2)	4.074 (0.344-8.184)	0.265
GG/GG	31 (11.6)	20 (12.4)	1.158 (0.576-2.33)	0.680
GG/GA	8 (3.0)	5 (3.1)	1.04 (0.313-3.452)	0.950
GG/AA	1 (0.4)	0 (0.0)	N/A	N/A
miR-34a rs2666433 G>A/miR-34a rs6577555 C>A				
GG/CC	74 (27.6)	44 (27.3)	1.000 (reference)	
GG/CA	68 (25.4)	30 (18.6)	0.737 (0.416-1.303)	0.293
GG/AA	11 (4.1)	14 (8.7)	2.062 (0.856-4.968)	0.107
GA/CC	70 (26.1)	40 (24.8)	0.938 (0.546-1.614)	0.818
GA/CA	26 (9.7)	26 (16.1)	1.67 (0.863-3.232)	0.128
GA/AA	1 (0.4)	1 (0.6)	1.728 (0.098-0.508)	0.709
AA/CC	17 (6.3)	6 (3.7)	0.6 (0.219-1.642)	0.320
AA/CA	1 (0.4)	0 (0.0)	N/A	N/A
AA/AA	0 (0.0)	0 (0.0)	N/A	N/A
miR-34a rs2666433 G>A/miR-130a rs731384 G>A				
GG/GG	114 (42.5)	68 (42.2)	1.000 (reference)	
GG/GA	36 (13.4)	18 (11.2)	0.852 (0.448-1.623)	0.627
GG/AA	3 (1.1)	2 (1.2)	1.156 (0.187-7.147)	0.876
GA/GG	74 (27.6)	49 (30.4)	1.119 (0.699-1.791)	0.641
GA/GA	23 (8.6)	17 (10.6)	1.308 (0.648-2.642)	0.454
GA/AA	0 (0.0)	1 (0.6)	N/A	N/A
AA/GG	14 (5.2)	6 (3.7)	0.749 (0.273-2.057)	0.576
AA/GA	4 (1.5)	0 (0.0)	N/A	N/A
AA/AA	0 (0.0)	0 (0.0)	N/A	N/A
miR-34a rs6577555 C>A/miR-130a rs731384 G>A				
CC/GG	121 (45.1)	69 (42.9)	1.000 (reference)	
CC/GA	38 (14.2)	19 (11.8)	0.865 (0.462-1.619)	0.651
CC/AA	2 (0.7)	2 (1.2)	1.745 (0.24-2.692)	0.583
CA/GG	73 (27.2)	41 (25.5)	0.975 (0.6-1.585)	0.918
CA/GA	22 (8.2)	15 (9.3)	1.189 (0.578-2.445)	0.637
CA/AA	0 (0.0)	0 (0.0)	N/A	N/A
AA/GG	8 (3.0)	13 (8.1)	2.881 (1.132-7.332)	**0.026**
AA/GA	3 (1.1)	1 (0.6)	0.627 (0.063-6.274)	0.691
AA/AA	1 (0.4)	1 (0.6)	1.825 (0.112-9.764)	0.673

Note: RIF, recurrent implantation failure; 95% CI, 95% confidence interval; AOR, adjusted odds ratio; AOR was adjusted by age ; N/A, not applicable.

Supplementary Table S3. Clinical variables in RIF patients stratified by miRNA polymorphisms status by ANOVA and Kruskal–Wallis test

Genotype	BMI (kg/m^2)	Homocysteine (μmol/L)	Folate (mg/ml)	Uric acid (mg/dl)	BUN (mg/dl)	Creatinine (mg/dl)	E2 (Basal) (pg/mL)	TSH (mU/L)	FSH (mU/L)
	mean±SD	mean±SD	mean±SD	mean±SD	mean±SD	mean±SD	mean±SD	mean±SD	mean±SD
miR-218-2 rs11134527 G>A									
AA	20.98±2.55	6.52±2.24	13.19±7.96	3.98±0.86	11.23±3.11	0.81±0.10	64.74±133.83	2.52±1.79	8.92±5.47
AG	21.69±3.51	6.66±1.56	16.14±6.94	3.91±1.00	10.18±2.84	0.78±0.10	57.19±104.57	2.08±1.23	9.10±3.56
GG	21.46±3.27	7.22±1.41	14.74±9.72	4.28±1.08	10.28±2.33	0.77±0.08	64.93±117.51	2.15±1.39	10.59±7.93
P^a	0.470	0.443	0.443	0.417	0.228	0.255	0.934	0.339	0.325[b]
miR-34a rs2666433 G>A									
GG	21.48±3.16	6.73±1.64	15.56±7.86	3.99±1.01	10.78±2.93	0.79±0.09	65.32±121.13	2.38±1.62	9.15±3.67
GA	21.61±3.32	6.66±1.95	14.56±8.83	4.02±0.95	10.13±2.80	0.78±0.10	54.80±109.07	1.88±0.90	9.13±5.99
AA	18.89±1.08	7.93±1.85	9.11±4.40	0.00±0.00	12.00±2.83	0.85±0.07	38.27±5.29	4.62±2.48	12.64±8.18
P^a	0.136	0.500	0.429	0.894	0.388	0.552	0.831	**0.012**[b]	0.479
miR-34a rs6577555 C>A									
CC	21.33±3.20	7.17±2.07	14.02±8.38	4.00±0.96	10.52±3.06	0.78±0.11	52.40±82.55	2.51±1.54	9.33±5.31
CA	21.80±3.42	6.21±1.29	15.62±7.99	4.09±1.00	10.63±2.77	0.80±0.09	62.15±126.60	1.94±1.24	8.90±4.47
AA	20.73±2.26	7.18±1.56	15.32±8.55	3.66±0.98	10.00±2.21	0.77±0.05	95.13±195.13	1.44±0.98	9.64±4.19
P^a	0.469	0.079	0.808	0.488	0.827	0.585[b]	0.421	**0.020**	0.872
miR-130a rs731384 G>A									
GG	21.61±3.32	6.64±1.49	15.25±8.23	3.94±0.96	10.34±2.55	0.80±0.10	65.29±128.72	2.25±1.52	9.40±4.98
GA	20.85±2.83	6.99±2.51	13.51±8.01	4.32±0.98	11.25±3.98	0.75±0.08	46.11±52.73	2.12±1.19	8.70±4.88
AA	21.33±2.53	8.80±0.00	8.96±0.00	2.80±0.42	10.07±0.83	0.73±0.06	38.97±2.30	2.15±1.25	9.02±3.46
P^a	0.464	0.405	0.659	0.067	0.837[b]	0.051	0.696	0.922	0.832

Genotype	LH (mU/L)	Prolactin (ng/mL)	WBC (10^3/μl)	Hgb (g/dl)	CD3(pan T)	CD4(helper T)	CD8(suppressor T)	CD19(B-Cell)	CD56(NK cell)
	mean±SD	mean±SD	mean±SD	mean±SD	mean±SD	mean±SD	mean±SD	mean±SD	mean±SD
miR-218-2 rs11134527 G>A									
AA	4.88±2.67	16.09±8.30	7.14±3.40	12.37±1.46	65.66±8.94	36.15±9.80	26.08±7.54	12.53±4.30	18.36±9.80
AG	4.73±2.31	12.72±5.05	7.04±2.35	12.64±1.39	67.03±12.48	34.17±9.21	29.88±8.51	11.41±5.27	17.53±8.95
GG	5.67±2.26	12.59±9.91	8.78±3.05	12.39±1.59	72.37±6.70	40.43±7.36	26.15±5.46	11.13±3.67	15.12±7.64
P[a]	0.403	0.104[b]	0.105	0.605	0.123	0.052	**0.047**	0.485	0.372
miR-34a rs2666433 G>A									
GG	4.59±2.02	14.95±7.37	7.16±2.64	12.64±1.41	66.64±9.52	35.80±9.20	27.63±7.74	11.60±5.48	17.79±9.04
GA	5.28±2.71	12.27±6.79	7.51±3.10	12.42±1.49	67.79±12.19	35.32±9.26	29.03±8.51	12.21±3.92	16.83±8.44
AA	5.79±4.95	10.01±4.57	5.50±1.65	11.97±0.81	71.52±14.77	36.98±12.57	27.48±7.40	8.67±2.67	18.23±15.29
P[a]	0.476[b]	0.134	0.444	0.569	0.558	0.909	0.659	0.147[b]	0.780[b]
miR-34a rs6577555 C>A									
CC	4.87±2.72	13.61±6.48	7.07±2.75	12.49±1.37	68.36±10.09	34.48±9.59	30.02±7.27	10.63±3.64	18.13±9.61
CA	5.17±2.15	12.02±6.27	7.61±3.00	12.51±1.59	65.71±13.12	37.13±8.30	25.64±8.41	13.62±5.49	16.05±7.96
AA	4.41±1.14	19.48±11.12	7.32±2.90	12.78±1.25	67.64±7.96	37.00±10.75	27.09±8.66	11.23±6.01	18.04±9.32
P[a]	0.633	**0.022**	0.639	0.816	0.512	0.344	**0.027**	**0.023[b]**	0.420
miR-130a rs731384 G>A									
GG	4.90±2.48	14.04±7.71	7.12±2.66	12.62±1.30	66.93±11.88	36.34±9.58	27.57±7.95	11.81±4.76	17.43±9.33
GA	4.68±2.00	12.22±4.57	7.49±2.79	12.23±1.66	69.54±7.74	34.08±8.47	30.18±8.28	11.83±4.96	16.32±7.77
AA	7.52±4.04	13.42±1.65	9.91±7.17	12.43±2.89	62.33±2.52	30.00±7.94	29.67±7.64	7.67±3.21	28.00±1.00
P[a]	0.283	0.631	0.780[b]	0.561[b]	0.420	0.326	0.345	0.338	0.101

Note: ANOVA, analysis of variance; BMI, body mass index; BUN, blood urea nitrogen; E2, estradiol; TSH, thyroid-stimulating hormone; FSH, follicle stimulating hormone; LH, luteinizing hormone; WBC, white blood cell; Hgb, hemoglobin; SD, standard deviation. [a]Calculated using ANOVA. [b]Calculated using the Kruskal-Wallis test.

Supplementary Table S4. Interaction analysis of the miRNA genotypes and characteristics of RIF among individual risk factors

Characteristics	*miR-34a rs6577555 C>A* CC		*miR-34a rs6577555 C>A* CA+AA	
	AOR(95% CI)*	*P*	AOR(95% CI)*	*P*
BMI (kg/m2)				
<24.2	1.000 (reference)		0.814 (0.45-1.474)	0.498
≥24.2	0.443 (0.181-1.087)	0.075	1.081 (0.313-3.73)	0.902
PT (s)				
<11.7	1.000 (reference)		**1.731 (1.037-2.892)**	**0.036**
≥11.7	**4.195 (1.724-0.212)**	**0.002**	**4.334 (1.462-2.846)**	**0.008**
aPTT (s)				
<33	1.000 (reference)		1.173 (0.518-2.659)	0.702
≥33	1.173 (0.518-2.659)	0.702	2.299 (0.793-6.671)	0.126
PLT (103/µl)				
<301	1.000 (reference)		1.141 (0.721-1.806)	0.573
≥301	1.127 (0.521-2.435)	0.762	**2.647 (1.111-6.306)**	**0.028**
Homocysteine (µmol/L)				
<8.7	1.000 (reference)		0.674 (0.176-2.579)	0.564
≥8.7	1.001 (0.092-0.894)	0.999	0.474 (0.038-5.849)	0.560
Uric acid (mg/dl)				
<5	1.000 (reference)		**2.296 (1.211-4.354)**	**0.011**
≥5	1.685 (0.586-4.843)	0.333	1.931 (0.572-6.523)	0.289
BUN (mg/dl)				
<12.6	1.000 (reference)		1.248 (0.711-2.192)	0.440
≥12.6	**2.521 (1.033-6.155)**	**0.042**	**4.818 (1.36-7.063)**	**0.015**
Creatinine (mg/dl)				
<0.9	1.000 (reference)		1.545 (0.879-2.716)	0.130
≥0.9	**4.968 (1.776-3.902)**	**0.002**	1.614 (0.609-4.282)	0.336
E2 (Basal) (pg/mL)				
<48.5	1.000 (reference)		1.055 (0.59-1.886)	0.857
≥48.5	**3.9 (1.041-4.609)**	**0.043**	2.405 (0.787-7.348)	0.124
TSH (mU/L)				
>0.849	1.000 (reference)		1.071 (0.563-2.037)	0.834
≤0.849	**0.096 (0.021-0.443)**	**0.003**	1.66 (0.488-5.639)	0.417
FSH (mU/L)				
<11.41	1.000 (reference)		1.013 (0.56-1.833)	0.965
≥11.41	2.016 (0.67-6.069)	0.212	**3.899 (1.094-3.901)**	**0.036**
LH (mU/L)				
<6.48	1.000 (reference)		0.978 (0.537-1.784)	0.943
≥6.48	**5.629 (1.504-1.07)**	**0.010**	3.248 (0.921-1.453)	0.067
WBC (103/µl)				
<9.8	1.000 (reference)		0.997 (0.613-1.621)	0.989
≥9.8	0.578 (0.222-1.506)	0.262	1.711 (0.746-3.924)	0.205
Hgb (g/dl)				
>11.2	1.000 (reference)		1.154 (0.712-1.87)	0.560
≤11.2	1.05 (0.464-2.376)	0.907	1.507 (0.608-3.738)	0.376

Note: NOTE: BMI, body mass index; PT, prothrombin time; aPTT, activated partial thromboplastin time; PLT, platelet count; BUN, blood urea nitrogen; E2, estradiol; TSH, thyroid-stimulating hormone; FSH, follicle stimulating hormone; LH, luteinizing hormone; WBC, white blood cell; Hgb, hemoglobin; 95% CI, 95% confidence interval; AOR, adjusted odds ratio; N/A, not applicable; AOR was adjusted by age. Folate, TSH and Hgb were lower 15% cut-off each level in RIF patients and controls; BMI, PT, aPTT, PLT, Homocysteine, Uric acid, BUN, Creatinine, E2, FSH, LH, and WBC were upper 15% cut-off each level in RIF patients and controls.

Supplementary Table S5. The area under the ROC curve of miRNA polymorphisms with clinical factors

Parameter	AUC	P value	95% CI
TSH>0.849mU/L	0.516	0.664	0.440-0.592
TSH≤0.849mU/L	0.852	**<0.0001**	0.675-0.954
BUN<12.6mg/dl	0.539	0.334	0.471-0.606
BUN≥12.6mg/dl	0.567	0.4845	0.399-0.724
PT<11.7s	0.576	0.0379	0.512-0.638
PT≥11.7s	0.507	0.941	0.356-0.657

Note: ROC, receiver operating characteristic; AUC, area under curve.

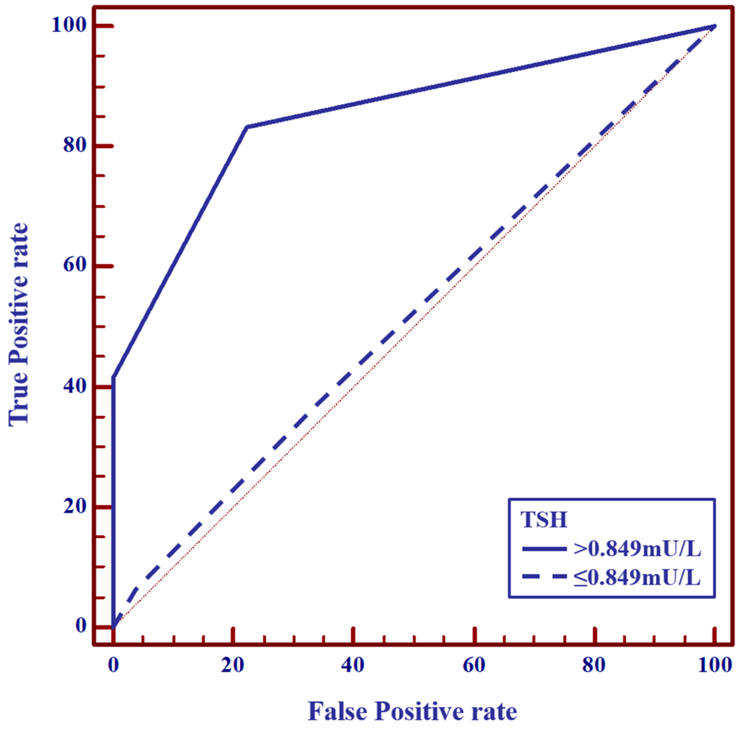

Figure S1. TSH level-dependent receiver operating characteristic (ROC) curve analysis of miR-34a rs6577555 C>A and RIF risk. For each independent ROC curve, computation was performed according to the thyroid-stimulating hormone (TSH) range. The ROC curve that incorporated miR-34a rs6577555 C>A and elevated TSH levels demonstrated a superior predictive value (AUC = 0.852) compared to the curve that solely considered TSH levels within the normal range (AUC = 0.516).

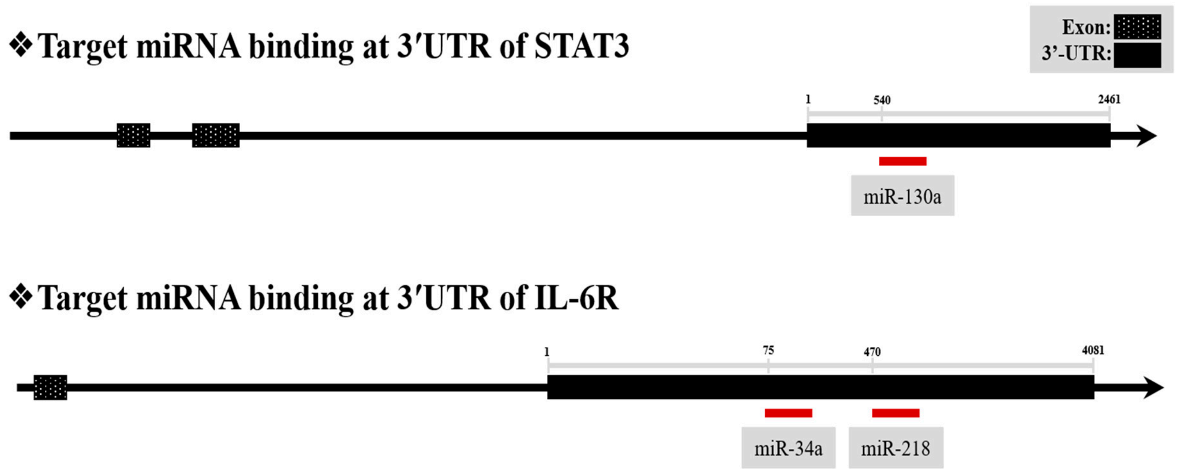

Figure S2. Predicted miRNA binding sites in the 3′-UTR of STAT3 and IL-6R. The schematic overview illustrates the 3' UTR of STAT3 and IL-6R, highlighting the miRNA binding sites along with the positions of the target genes.
